# Suppression of Lipid Accumulation by Indole-3-Carbinol Is Associated with Increased Expression of the Aryl Hydrocarbon Receptor and CYP1B1 Proteins in Adipocytes and with Decreased Adipocyte-Stimulated Endothelial Tube Formation

**DOI:** 10.3390/ijms17081256

**Published:** 2016-08-03

**Authors:** Mei-Lin Wang, Shyh-Hsiang Lin, Yuan-Yu Hou, Yue-Hwa Chen

**Affiliations:** 1 School of Nutrition and Health Sciences, Taipei Medical University, 250 Wu-Hsing Street, Taipei 110, Taiwan; b8706083@tmu.edu.tw (M.-L.W.); lin5611@tmu.edu.tw (S.-H.L.); 2 Department of Food and Beverage Management, Mackay Medicine, Nursing and Management College, Taipei 112, Taiwan; s212@eip.mkc.edu.tw; 3 Cancer Research Center, Taipei Medical University Hospital, Taipei Medical University, 250 Wu-Hsing Street, Taipei 110, Taiwan

**Keywords:** indole-3-carbinol, obesity, adipocytes, adipogenesis, angiogenesis, aryl hydrocarbon receptor

## Abstract

This study investigated the effects of indole-3-carbinol (I3C) on adipogenesis- and angiogenesis-associated factors in mature adipocytes. The cross-talk between mature adipocytes and endothelial cells (ECs) was also explored by cultivating ECs in a conditioned medium (CM) by using I3C-treated adipocytes. The results revealed that I3C significantly inhibited triglyceride accumulation in mature adipocytes in association with significantly increased expression of AhR and CYP1B1 proteins as well as slightly decreased nuclear factor erythroid-derived factor 2–related factor 2, hormone-sensitive lipase, and glycerol-3-phosphate dehydrogenase expression by mature adipocytes. Furthermore, I3C inhibited CM-stimulated endothelial tube formation, which was accompanied by the modulated secretion of angiogenic factors in adipocytes, including vascular endothelial growth factor, interleukin-6, matrix metalloproteinases, and nitric oxide. In conclusion, I3C reduced lipid droplet accumulation in adipocytes and suppressed adipocyte-stimulated angiogenesis in ECs, suggesting that I3C is a potential therapeutic agent for treating obesity and obesity-associated disorders.

## 1. Introduction

Obesity has been a major concern since the 20th century, and its prevalence is increasing in many countries. In addition to affecting the physical and psychological status of obese people, excess weight, mainly accumulated in the form of lipid in the adipocytes of white adipose tissue (WAT), considerably increases the risk of developing various chronic diseases, including diabetes, cardiovascular diseases, and cancers [1]. Moreover, in addition to mature adipocytes, WAT consists of fibroblastic preadipocytes, endothelial cells (ECs), and macrophages. The expansion of mature adipoctyes, the differentiation of preadiopocytes to adipocytes, the formation of new vessels of ECs, and the infiltration of macrophages accompany the progress of WAT expansion in obesity [2]. Mature adipocytes are not only a lipid storage site but also produce and secrete different adipokines and factors such as leptin, interleukin (IL)-6, and vascular endothelial growth factor (VEGF), which are closely associated with angiogenesis and other pathological conditions in obesity [2,3]. Therefore, the massive accumulation of lipids in mature adipocytes in obesity causes intimate interactions between mature adipocytes and adjacent cells, including vascular ECs in WAT, which may contribute to the pathological characteristics of obesity, including different metabolic disorders and cancers [4,5,6].

Indole-3-carbinol (I3C) is a bioactive indolic compound derived from glucosinolates in cruciferous vegetables, such as broccoli, cabbage, Brussels sprouts, kale, and cauliflower [7]. I3C possesses anticarcinogenic activities [7,8,9]. Recently, I3C has shown to exhibit antiobesity activity by reducing body weight and fat in animals fed a high-fat diet [10,11] and by inhibiting the differentiation of 3T3-L1 preadipocytes [12]. Being an activator of the aryl hydrocarbon receptor (AhR), a ligand-activated transcription factor crucial in adipogenesis and angiogenesis [13,14,15,16], we considered that I3C executes its activities through the AhR. Although I3C has been shown to inhibit the differentiation of preadipocytes, knowledge regarding its effects on lipid accumulation in mature adipocytes and on adipocyte-associated angiogenesis is limited. Because increased triglyceride (TG) accumulation in mature adipocytes is positively associated with obesity and associated metabolic disorders, compounds that reduce the TG content in adipocytes may have therapeutic roles in obesity and related pathological disorders. This study was aimed at examining the roles of I3C in the adipogenesis of mature adipocytes and in the cross-talk between mature adipocytes and ECs. Furthermore, the effects of I3C on factors associated with AhR-mediated pathways were determined. Results obtained in this study may facilitate elucidating the potential use of I3C in adjunctive treatment for obesity and associated disorders.

## 2. Results

### 2.1. Effects of I3C on Cell Viability and Lipid Accumulation in Mature Adipocytes

**Figure 1:**
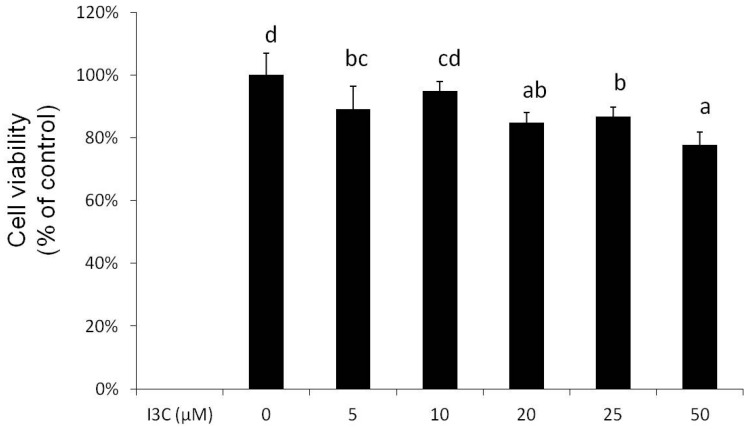
Effect of indole-3-carbinol (I3C) on cell viability in differentiated adipocytes. Cells were treated with various concentrations of I3C for 24 h, and cell viability was measured using an MTS assay kit. Values are the mean ± SD from three measurements. a–d, bars with different letters significantly differ from each other (*p* < 0.05).

**Figure 2:**
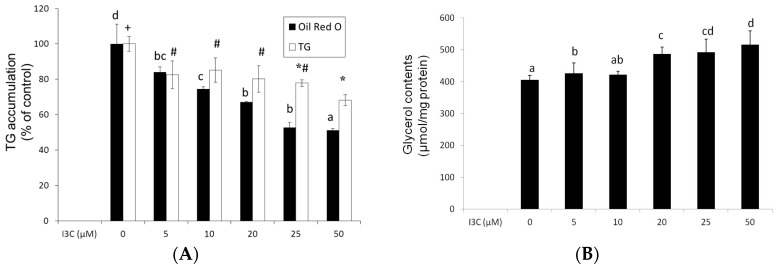
Effect of indole-3-carbinol (I3C) on lipid accumulation in differentiated adipocytes. Cells were treated with various concentrations of I3C for 24 h, and the cells were stained with oil red O. Intracellular oil red O and triglyceride (TG) (**A**) and extracellular glycerol (**B**) contents were quantified using the method described in the Materials and Methods. Values are the mean ± SD from three measurements. Bars with different letters (a–d) or different symbols (+, #, *) significantly differ from each other (*p* < 0.05).

At concentrations of 5–50 μM, I3C slightly, in a concentration-dependent manner, reduced (5%–22%) the viability of mature adipocytes after 24 h of treatment (Figure 1), and these concentrations were used for the following experiments. Results from the oil red O staining and analysis of intracellular TG content revealed that I3C concentration-dependently reduced lipid accumulation in the mature adipocytes (Figure 2A), and this reduction was associated with the increased release of glycerol by mature adipocytes (Figure 2B).

### 2.2. Effects of I3C on Adipocyte-Induced Tube Formation in ECs

**Figure 3:**
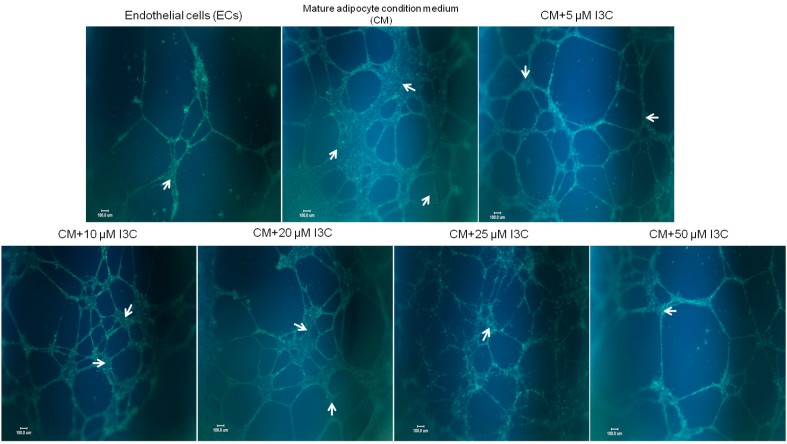
Effects of indole-3-carbinol (I3C) on tube formation in endothelial cells activated with the conditioned medium (CM) from mature adipocytes. Following 6 d differentiation, mature adipocytes were treated with I3C for 24 h, and the CM was collected and used to cultivate endothelial EA hy926 cells, which were grown on Matrigel-coated plates for 24 h. Formation of tube-like structures (as indicated in arrows) was observed and photographed under a microscope after staining with calcein AM fluorescent dye. Pictures are representative of three independent experiments. Scale Bars = 100 μm.

**Figure 4:**
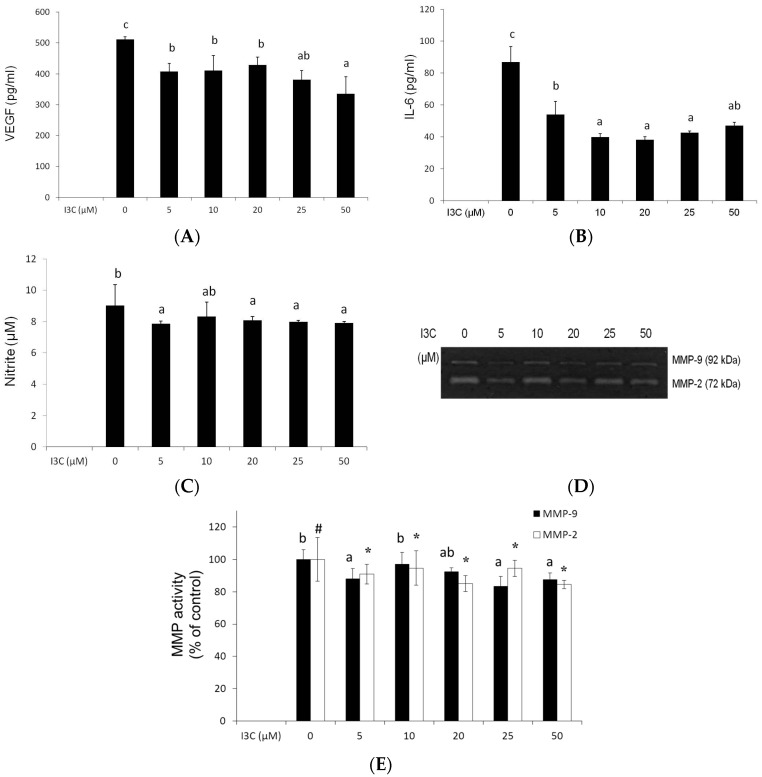
Effects of indole-3-carbinol (I3C) on the angiogenic factors in the cultured medium from differentiated adipocytes. Following differentiation, adipocytes were treated with various concentrations of I3C in Dulbecco’s modified Eagle’s medium containing 1% fetal bovine serum for 24 h. The medium was retrieved for analyzing the vascular endothelial growth factor (VEGF) (**A**); interleukin (IL)-6 (**B**); nitric oxide (NO) (**C**); and matrix metalloproteinase (MMP) activities (**D**); and quantification (**E**) by using the methods described in the Materials and Methods. Values are the mean ± SD from three measurements, and bars with different letters (a–c) or different symbols (#,*) significantly differ from each other (*p* < 0.05).

Expansion of adipose tissue is accompanied by increased endothelial angiogenesis; therefore, endothelial tube formation was used to explore the effects of I3C on interactions between mature adipocytes and ECs. The results revealed that the conditioned medium (CM) from adipocytes significantly stimulated the formation of tube-like endothelial structures; however, the presence of I3C reduced the interconnection networks among ECs (Figure 3), and the suppression was accompanied by the decreased production of proangiogenic factors, including VEGF (Figure 4A), IL-6 (Figure 4B), and to a lesser extent, NO (Figure 4C) and matrix metalloproteinases (MMPs) (Figure 4D,E), by the mature adipocytes.

### 2.3. Effects of I3C on Protein Expression by Mature Adipocytes

**Figure 5:**
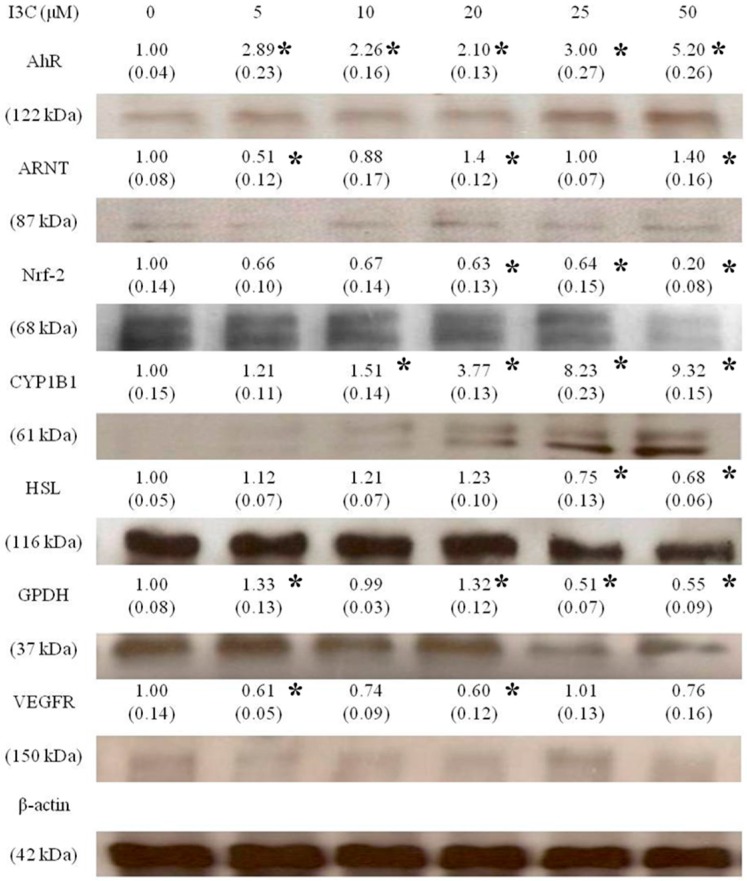
Effects of I3C on the aryl hydrocarbon receptor (AhR), AhR nuclear translocator (ARNT), CYP1B1, hormone-sensitive lipase (HSL), glycerol-3-phosphate dehydrogenase (GPDH), nuclear factor erythroid-derived factor 2-related factor 2 (Nrf-2), and vascular endothelial growth factor receptor (VEGFR) protein expression in differentiated adipocytes. Mature adipocytes were treated with various concentrations of I3C for 24 h, and proteins were retrieved and subsequently measured using a Western blot analysis. The expression of each protein was adjusted using β-actin, and values represented are a fold of the control. The values in parentheses represent standard deviations from three different measurements in this experiment. * indicates significant different from the negative control (*p* < 0.05).

To determine the effects of I3C on the expression of AhR-, adipogenesis-, and angiogenesis-associated proteins by mature adipocytes, a Western blot analysis was performed. Figure 5 shows that I3C significantly enhanced the expression of the AhR and the gene that it regulates, CYP1B1, in mature adipocytes, but only slightly enhanced ARNT expression at high concentrations. In contrast, the expression of nuclear factor erythroid-derived factor 2-related factor 2 (Nrf-2), HSL, GPDH, and VEGFR was slightly downregulated by I3C, especially at higher concentrations. The original blots for each protein are shown in the Figure S1.

## 3. Discussion

In the present study, we demonstrated for the first time that a cruciferous vegetable bioactive component, I3C, at concentrations of 25–50 μM, significantly reduced TG accumulation in mature adipocytes, and this effect was associated with increased expression of the AhR and CYP1B1 proteins in adipocytes. In addition, I3C suppressed the production of proangiogenic mediators by mature adipocytes, leading to decreased endothelial tube formation stimulated by the adipocytes. Obesity is positively associated with various metabolic disorders and cancers, and the expansion of WAT accompanied by enlarged adipocytes is believed to be a key event in these pathological conditions involving the adipogenesis and angiogenesis of adipose tissues. Because the mature adipocyte is the major cell type in the WAT of obese people, the suppressive effects of I3C on TG accumulation and stimulated endothelial tube formation suggest that I3C has potential therapeutic effects on obesity, and possibly, on obesity-associated metabolic disorders.

Several studies have indicated the protective effects of I3C on cancers and obesity; these effects are believed to be intimately associated with the AhR-mediated pathways. The liganded AhR dimerizes with the ARNT to modulate various pathways, including carcinogen metabolism and adipocyte differentiation. The AhR ligands, such as 2,3,7,8-tetrachlorodibenzo-p-dioxin (TCDD), β-naphthoflavone (BNF), and polychlorinated biphenyl, activate the transcription of xenobiotic metabolizing enzymes CYP1A and CYP1B [17]. On the other hand, the AhR negatively regulates adipocyte differentiation [13,18], and TCDD suppresses adipocyte differentiation [19,20], whereas the AhR antagonist α-naphthoflavone (ANF) increases lipid accumulation in mature adipocytes [21]. I3C is a naturally occurring AhR agonist that exhibits antiobesity activities, such as the reduction of body and WAT weights in high-fat-diet-induced obese mice and the inhibition of adipocyte differentiation by activating the silent mating type information regulation 2 homolog 1 and subsequently downregulating the expression of PPARγ2, C/EBPα, and aP2, factors crucial for differentiation [12,22]. In this study, we observed that I3C may act through activating lipolysis and/or inhibiting lipogenesis to reduce TG accumulation in mature adipocytes from the evidence that I3C increased the glycerol released into the medium and suppressed the expression of GPDH, a key enzyme in lipogenesis, at high concentrations. The generation of glycerol from TG hydrolysis acts through sequential actions of different lipases, including adipose TG lipase (ATGL), HSL, and monoglyceride lipase, and both HSL and ATGL are major enzymes involved in TG catabolism in adipose tissue [23,24]. I3C might not act chiefly through HSL activation to increase lipolysis in mature adipocytes, because the expression of the HSL protein was slightly reduced by I3C. However, the phosphorylation status of HSL was not determined, so the detailed mechanisms require investigated further.

Not only by inhibiting adipocyte differentiation does AhR regulate lipid metabolism. AhR is a constitutive inhibitor of TG synthesis in mouse embryo fibroblasts (MEFs) [25]; in addition, a transient fatty liver was observed in AhR-null mice [26], suggesting that AhR acts as a suppressor of lipid accumulation. Because we observed that I3C enhanced the expression of the AhR protein in adipocytes, this may be also involved in the suppressed TG accumulation in adipocytes by I3C. Similarly, Tano et al. [27] indicated that BNF represses the expression of enzymes in the fatty acid synthesis pathway in primary hepatocytes, leading to a decrease in fatty acid production, and these effects are dependent upon AhR. TCDD increased the expression of lipolysis-associated factors in treated mice, and this increase is related to the TCDD-induced wasting syndrome [28,29]. On the other hand, we previously reported that the AhR antagonist ANF reduced the expression of the AhR in association with increased TG accumulation in adipocytes [21]. Therefore, we hypothesize that I3C reduces lipid accumulation in adipocytes by inducing AhR expression and then reducing lipogenesis and increasing lipolysis responses in 3T3-L1 adipoyctes.

Nrf-2 is another transcription factor crucial in the expression of xenobiotic metabolism enzymes and in adipogenesis [30]. An intimate interaction between Nrf-2 and the AhR pathways exists. Miao et al. [31] reported that TCDD induces Nrf-2 expression by activating AhR-XRE binding in Hepa1c1c cells. Conversely, Nrf-2−/− MEFs had low levels of AhR expression, and the Nrf-2 activator upregulated the AhR pathways, subsequently inhibiting adipogenesis in Nrf-2+/+ MEFs. Furthermore, stable knockdown of Nrf-2 in 3T3-L1 cells inhibited enhanced adipogenesis caused by the deficiency of kelch-like ECH-associated protein 1 [32], indicating the suppressive role of Nrf-2 in adipogenesis [25]. However, not only I3C but also the AhR antagonist ANF suppressed Nrf-2 protein expression in mature adipocytes. These results suggest that Nrf-2 predominantly affects adipocyte differentiation; however, it either does not regulate or only slightly regulates AhR expression and angiogenesis in mature adipocytes. This mechanism requires further investigation.

In addition to reducing lipid accumulation in mature adipocytes, I3C (5–50 μM) inhibited endothelial tube formation stimulated by the CM from mature adipocytes, and this suppression was associated with the decreased secretion of angiogenic factors, including VEGF, IL-6, NO, and MMPs, by mature adipocytes. Substantial tissue remodeling that occurs within adipose tissues during fat mass expansion is accompanied by angiogenesis [33,34]. WAT not only is a TG storage depot but also acts as an endocrine organ because of its abilities to produce and secrete various adipokines, growth factors, and inflammatory mediators, which may alter the functions of different cells. Higher serum levels of proinflammatory mediators, such as C-reactive protein, IL-6, and tumor necrosis factor α, as well as angiogenic factors, including VEGF, IGF-1, MMPs, and leptin, were observed in obese people compared with those in healthy people [15], and these factors are involved in the pathological changes of obesity. Mature human adipose tissue extract produces numerous angiogenic factors and induces endothelial tube formation [35]. The expression of VEGF positively correlates to the size of adipocytes [36], and is enhanced by IL-6 [37]. Because I3C was observed to reduce IL-6 expression in the WAT in high-fat-diet-induced obese mice [11] and in LPS-stimulated macrophages [38], we hypothesize that I3C (5–50 μM) suppresses adipocyte-induced angiogenesis both by reducing TG accumulation in mature adipocytes leading to reduce the secretion of angiogenic mediators, and by directly inhibiting IL-6 expression in mature adipocytes. Alternatively, I3C lowers leptin levels and increases serum adiponectin levels in obese animals [10]; these effects may contribute to the antiangiogenic effect of I3C observed in this study. Thus, I3C can not only diminish adipocyte differentiation but also eliminate angiogenesis, thereby inhibiting obesity.

The estimated daily intake is at the equivalent of 6.4 mg of I3C in the UK, where the cruciferous vegetable tends to be a dietary staple [39]. Ideally, this dose would generate ca. 9 μM of plasma I3C concentration in a 70 kg subject on the basis that blood volume comprises 7% of the body weight without considering digestion and absorption. Similarly, the plasma I3C peaked concentration was 4.13 μg/mL (ca. 28 μM) after orally administering 250 mg/kg of I3C to mice [40]. Although the plasma I3C concentration from ordinary vegetable consumption is lower than the concentrations we used in this study, higher plasma concentrations may be possibly achieved by taking I3C dietary supplements. Alternatively, several acid-catalyzed compounds formed following oral consumption of I3C have been identified and may contribute to the protective roles of I3C [41,42,43]. Among these derivatives, 3,3′-diindolylmethane (DIM) has been reported to play a protective role in metabolic diseases, including reducing blood glucose and increasing antioxidative enzymes in diabetic mice [44], as well as in alleviating hepatic steatosis and inflammation in Nonalcoholic steatohepatitis (NASH) mouse models [45]. Furthermore, a significant amount of DIM could be detected in the plasma and various organs after oral administration of I3C [40]. In addition, different studies have indicated that orally giving 400 or 800 mg/day of I3C to human subjects for up to 4.8 years showed no adverse effects [46,47]. Finally, although the AhR has been associated with carcinogenesis and toxicity, most studies suggest that I3C acts as a chemopreventive agent. Because I3C decreases body weight, reduces adipocyte lipid accumulation, and because I3C derivatives possess protective effects in metabolic disorders associated with obesity, the potential use of I3C in treating obesity or obesity-associated diseases is plausible, although the effects of I3C in vivo require further investigation.

## 4. Materials and Methods

### 4.1. Chemicals and Biochemicals

I3C, insulin, dexamethasone (Dex), 3-isobutyl-1-methyl-xanthine (IBMX), and dimethyl sulfoxide (DMSO) were purchased from Sigma Chemical (St. Louis, MO, USA). Dulbecco’s modified Eagle’s medium (DMEM), sodium bicarbonate, fetal bovine serum (FBS), calf serum, trypan blue, and trypsin were obtained from GIBCO BRL (Grand Island, NY, USA). I3C was dissolved in DMSO, and the final concentration of DMSO in culture media was 0.1% (*v*/*v*).

### 4.2. Cell Culture

The murine preadipocyte cell line 3T3-L1, which is typically used as a model for studying adipocyte differentiation and biology, was purchased from the Bioresource Collection and Research Center (BCRC #60159; Hsinchu, Taiwan). The cells were grown in a monolayer in DMEM supplemented with 10% fetal bovine serum (FBS) at 37 °C in a 95% air and 5% CO_2_ environment. To induce adipocyte differentiation, 3T3-L1 preadipocytes were cultivated in a DMEM differentiation medium that contained 0.25 μM Dex, 10 μg/mL insulin, and 0.5 mM IBMX for 2 days and then maintained in insulin-containing DMEM for another 4 days to obtain round mature adipocytes. After the medium was replaced with DMEM containing 1% FBS, the mature adipocytes were treated with I3C for 24 h, and the cells and the medium were analyzed.

To examine tube formation, a human endothelium-derived cell line with vascular EC characteristics, EA hy926, was used. EA hy926 cells were provided by Dr. Cora-Jean Edgell (University of North Carolina, Chapel Hill, NC, USA), who established and characterized the cells [48], which are now available from the American Type Culture Collection (ATCC^®^ CRL-2922). The cells were maintained in DMEM supplemented with 10% FBS at 37 °C in a 95% air and 5% CO_2_ environment.

### 4.3. Cytotoxicity

To determine the cytotoxic effects of I3C on mature adipocytes, cells were treated with different concentrations of I3C for 24 h, and cytotoxicity was evaluated by measuring the absorbance of the formazan product of 3-(4,5-dimethylthiazol-2-yl)-5-(3-carboxymethoxyphenyl)-2-(4-sulfophenyl)-2*H*-tetrazolium (MTS) produced by live cells by using a microplate reader at OD490 nm.

### 4.4. Lipid Accumulation and Glycerol Release of 3T3-L1 Adipocyte

To explore the effects of I3C on lipid accumulation, intracellular TG accumulation in mature adipocytes was determined using oil red O staining and was quantified using a commercial Randox TRIGS (Cat TG213) assay kit (Randox Labs, Crumlin, UK). Glycerol released into a medium, regarded as a marker of lipolysis, was analyzed using a commercial Randox glycerol (Cat GY105) assay kit.

### 4.5. Tube Formation Assay

To determine whether I3C affects the cross-talk between mature adipocytes and ECs, mature adipocytes were treated with I3C for 24 h, and the conditioned medium (CM) was retrieved and used for cultivating EA hy926 ECs, which were grown on BD Matrigel-coated plates for 24 h. The ECs were subsequently stained with calcein AM fluorescent dye (BD Biosciences, San Jose, CA, USA), and networks of vessel-like structures were observed and photographed using a fluorescent microscope.

### 4.6. Assays of Nitric Oxide, VEGF, IL-6, and Matrix Metalloproteinase Activities

To determine the effects of I3C on the production of angiogenic factors by mature adipocytes, the levels of VEGF, and IL-6 in the CM of mature adipocytes were determined using mouse DuoSet VEGF, and IL-6 commercial enzyme-linked immunosorbent assay systems (R & D System, Minneapolis, MN, USA), respectively. The amount of nitric oxide (NO) in the CM was determined using the Griess reagent. The activities of matrix metalloproteinase (MMP)-2 and -9 in the CM were determined through gelatin zymography, in which a culture medium containing 5 μg of protein was separated on a 10% SDS-PAGE gel that contained 1 mg/mL gelatin and then stained with 0.5% Coomassie Blue R-250. Clear bands in the destained gel against a blue background indicated the presence of MMP-2 and -9 (92 kDa) and were quantitated using Image-Pro Plus software (Media Cybernetics, Silver Spring, MD, USA).

### 4.7. Western Blot Analysis

Expression of proteins related to the AhR-mediated pathway, lipid metabolism, and angiogenesis in adipocytes was analyzed through Western blot. Following separation on a 10% SDS-PAGE gel, cellular proteins (15 μg), including AhR (1:1000, Santa Cruz Biotechnology, Santa Cruz, CA, USA), vascular endothelial growth factor receptor (VEGFR, 1:500, Santa Cruz Biotechnology), the AhR nuclear translocator (ARNT, 1:1000, Abcam Inc., Cambridge, MA, USA), CYP1B1 (1:1000, Abcam Inc.), Nrf2 (1:1000, Abcam Inc.), glycerol-3-phosphate dehydrogenase (GPDH, 1:1000, Abcam Inc.), hormone-sensitive lipase (HSL, 1:1000, Origene Technologies, Inc., Rockville, MD, USA) and β-actin (1:500, Novus Biologicals, Littleton, CO, USA), were electroblotted onto a polyvinylidene difluoride membrane and detected with specific protein monoclonal antibodies. Following the addition of peroxidase-conjugated immunoglobulin G (Millipore Corporation, Billerica, MA, USA) and detection with Amersham Enhanced Chemiluminescence™ western blotting detection reagents (GE Healthcare, Piscataway, NJ, USA), the specific proteins were quantitated using Image-Pro Plus software (Media Cybernetics, Silver Spring, MD, USA).

### 4.8. Statistical Analysis

Values are expressed as the mean ± standard deviation (SD). One-way analysis of variance followed by Fisher’s least significant difference test were performed using SAS software version 9.1 (SAS Institute, Cary, NC, USA) to compare the differences between groups. Differences were considered statistically significant at *p* < 0.05.

## 5. Conclusions

I3C significantly reduced lipid accumulation in mature adipocytes and suppressed adipocyte-stimulated tube formation in ECs, and these effects are associated with the decreased secretion of angiogenic factors by mature adipocytes, including VEGF, IL-6, and NO. In addition, I3C increased the expression of the AhR and CYP1B1 proteins in mature adipocytes. These results suggest that I3C can be potentially used for facilitating weight loss and alleviating obesity-associated disorders.

## Supplementary Materials

Supplementary materials can be found at http://www.mdpi.com/1422-0067/17/8/1256/s1.
