# Supplementary material for: Suppression of Lipid Accumulation by Indole-3-Carbinol Is Associated with Increased Expression of the Aryl Hydrocarbon Receptor and CYP1B1 Proteins in Adipocytes and with Decreased Adipocyte-Stimulated Endothelial Tube Formation
Source: Int J Mol Sci. 2016 Aug 3;17(8):1256. doi: 10.3390/ijms17081256 (PMC5000654; doi:10.3390/ijms17081256)
Supplement: Supplementary file 1 [file ijms-17-01256-s001.pdf]

Int. J. Mol. Sci. **2016**, *17*, 1256; doi:10.3390/ijms17081256

Supplementary Information: Suppression of Lipid Accumulation by Indole-3-Carbinol Is Associated with Increased Expression of the Aryl Hydrocarbon Receptor and CYP1B1 Proteins in Adipocytes and with Decreased Adipocyte-Stimulated Endothelial Tube Formation

Mei-Lin Wang, Shyh-Hsiang Lin, Yuan-Yu Hou and Yue-Hwa Chen

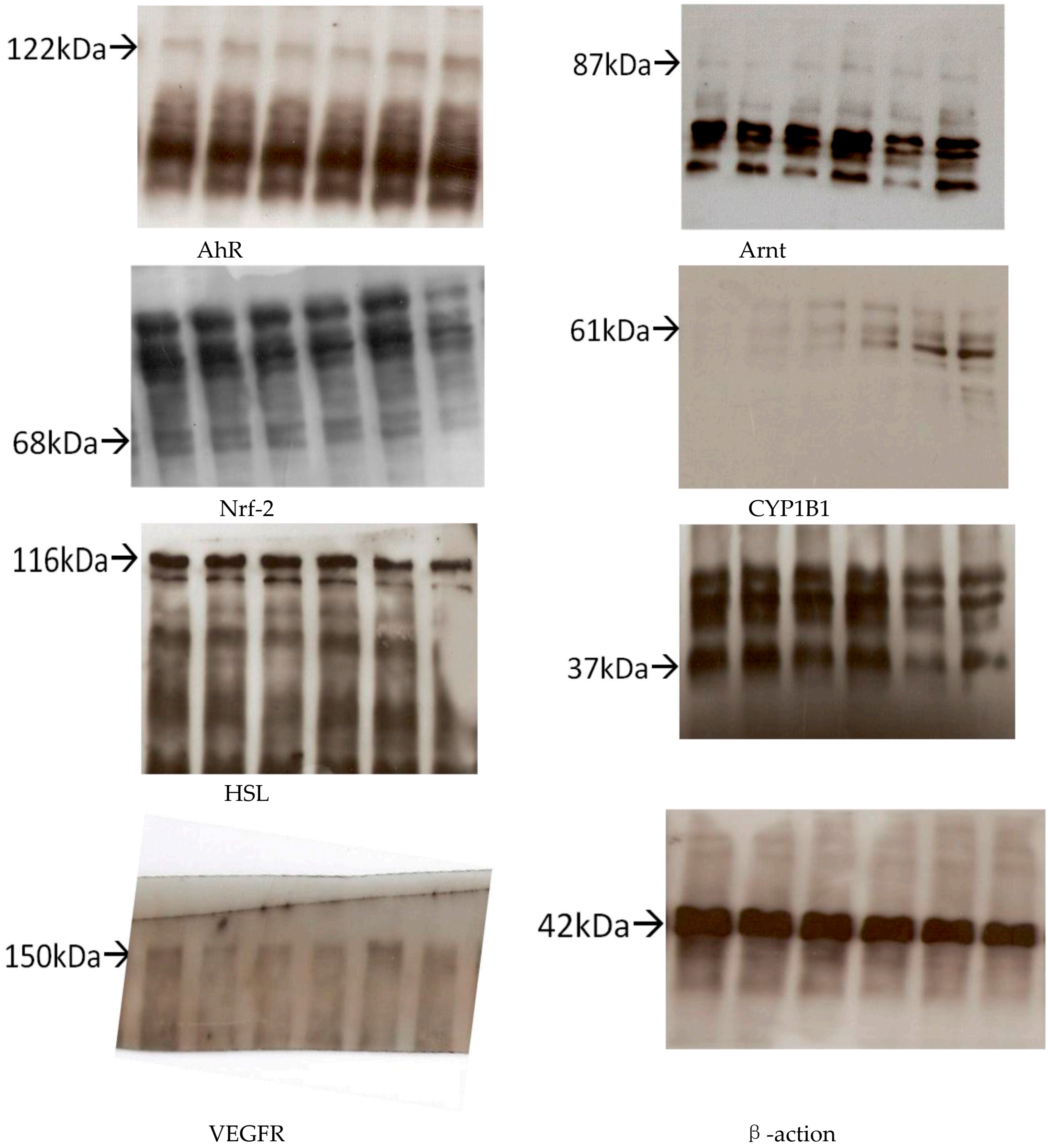

Figure S1. Original blots for each protein.
